# Dietary Inhibitors of CYP3A4 Are Revealed Using Virtual Screening by Using a New Deep-Learning Classifier

**DOI:** 10.1021/acs.jafc.2c00237

**Published:** 2022-02-01

**Authors:** Yelena Guttman, Zohar Kerem

**Affiliations:** Institute of Biochemistry, Food Science and Nutrition, The Robert H. Smith Faculty of Agriculture, Food and Environment, The Hebrew University of Jerusalem, POB 12, Rehovot 76100, Israel

**Keywords:** cytochrome P450 3A4 (CYP3A4), dietary compounds, food−drug interactions, deep learning, intestine

## Abstract

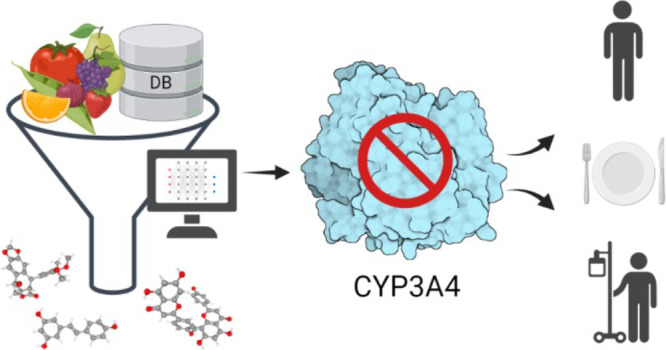

CYP3A4 is the main human enzyme responsible for phase I metabolism of dietary compounds, prescribed drugs and xenobiotics, steroid hormones, and bile acids. The inhibition of CYP3A4 activity might impair physiological mechanisms, including the endocrine system and response to drug admission. Here, we aimed to discover new CYP3A4 inhibitors from food and dietary supplements. A deep-learning model was built that classifies compounds as either an inhibitor or noninhibitor, with a high specificity of 0.997. We used this classifier to virtually screen ∼60,000 dietary compounds. Of the 115 identified potential inhibitors, only 31 were previously suggested. Many herbals, as predicted here, might cause impaired metabolism of drugs, and endogenous hormones and bile acids. Additionally, by applying Lipinski’s rules of five, 17 compounds were also classified as potential intestine local inhibitors. New CYP3A4 inhibitors predicted by the model, bilobetin and picropodophyllin, were assayed in vitro.

## 1 Introduction

Accumulated evidence points to the potent inhibition of cytochrome P450 3A4 (CYP3A4) by dietary phytochemicals, many of which are consumed as spices, dietary supplements, and herbal supplements.^1^ CYP3A4 is the main enzyme involved in the phase I metabolism of a wide range of endogenous compounds, i.e., steroid hormones, lipids, and bile acids, as well as xenobiotics, including dietary compounds and over 50% of prescribed drugs. The inhibition of CYP3A4 might cause various physiological consequences, such as cholestasis, a condition characterized by accumulation of toxic bile acids, impairment of the endocrine system signaling, and an increased risk of drug toxicity.^2−4^ However, deliberate inhibition of CYP3A4-mediated drug metabolism is sometimes utilized to increase the oral bioavailability of certain medications previously administered intravenously.^5^

The human CYP3A4 is recognized to be as active in the small intestine as it is in the liver. It accounts for approximately 80% of the total intestinal P450 content and represents the principal intestinal drug-metabolizing system in humans. Although the total amount of CYP3A expressed in the human small intestine represents approximately only 1% of the amount expressed in the liver, the substantial intestinal metabolism is due to prolonged exposure times.^6^ The predominance of CYP3A4 in the human intestine enables it to act several-fold more efficiently in the intestine than in the liver.^7^

Many CYP3A4 inhibitors in our diet belong to the large and diverse family of polyphenolics, including flavonoids, phenolic acids, phenolic alcohol, stilbenoids, and lignans.^1^ Data about plant-derived CYP3A4 inhibitors have accumulated slowly over the past 20 years. The discovery of new inhibitors has been limited by the time, resources, and compounds’ availability, needed for in vitro and in vivo assays. In recent years, chemoinformatic and machine-learning approaches have been used to identify the relationships between the structural and chemical properties of compounds and their biological activities. Several successful studies used in silico tools to predict CYP inhibitors.^8−11^ These methods allow the rapid and efficient virtual screening of large chemical databases for compounds with the activity of interest. While virtual screening is widely used to predict pharmaceutical synthetic inhibitors, e.g., of CYP3A4 and predict the outcome drug–drug interactions,^12,13^ screening dietary and food-derived compounds, which might induce food–drug and herb–drug interactions (FDI; HDI), is scarce. Here, we developed the tools to virtual screen comprehensive libraries of dietary compounds to discover new dietary CYP3A4 inhibitors. We used open-source in vitro data to design a ligand-based, deep-learning classifier and used it to identify potential CYP3A4 inhibitors. We present the prediction and discovery of novel CYP3A4 natural dietary inhibitors, which were identified by applying this predictive model.

## 2 Materials and Methods

### 2.1 Materials

Picropodophyllin (≥99.85%) was purchased from MedChemExpress (NJ, USA). Bilobetin (≥98%) was purchased from the Cayman Chemical Company (MI, USA).

### 2.2 Virtual Screening

**Figure 1:**
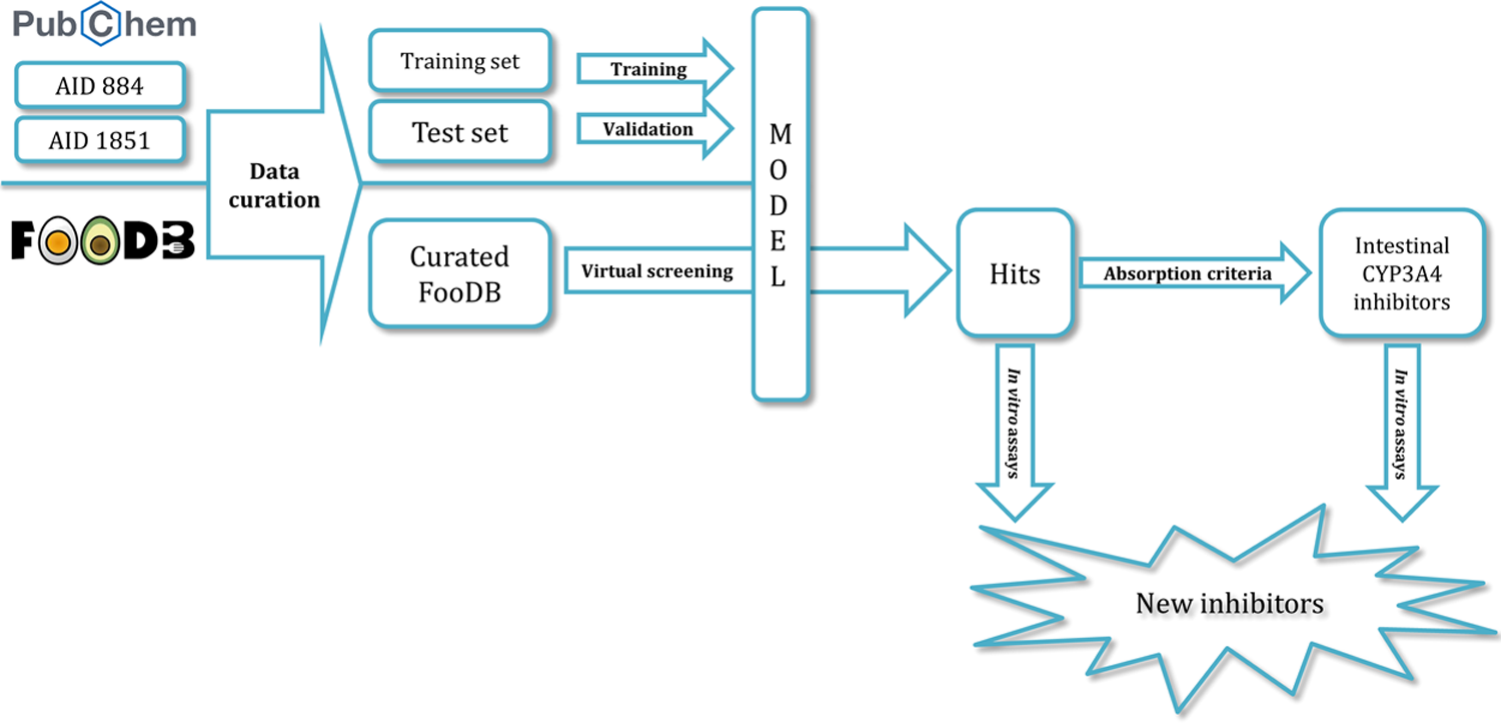
Study design.

A workflow in KNIME analytics platform 4.0.3^14^ was created to prepare and analyze the virtual screening. The general study design is summarized and presented in Figure 1.

#### 2.2.1 Dataset Preparation

AID884^15^ and AID1851^16^ datasets were downloaded from the PubChem BioAssay Database. Each dataset includes the results of a single high-throughput assay, measured as the dealkylation of luciferin-6′-phenylpiperazinylyl to luciferin by human CYP3A4. Luminescence indicative of luciferin generation decreased in the presence of inhibitors. In these high-throughput assays, compounds were added at graduated concentrations and the corresponding decrease in luminescence was used to determine the potency of each compound.

A KNIME workflow was created for data curation. Fragmented compounds were removed. Duplicates were excluded as follows: (i) in cases of duplicates with the same activity, only one entry was retained and (ii) in cases of duplicates with different biological activities, both entries were excluded. Overall, 1760 compounds with full dose-dependent response curves and efficacy levels of 80% greater than the respective controls (curve class -1.1) were classified as active. The potency of compounds classified as active was in the range of 0.032–15.85 μM. Overall, 8893 compounds were classified as inactive (curve class 4.0). The compounds were desalted in silico, and their protonation states at physiological pH (7.4) were determined using Epik (Schrödinger, NY, USA).

#### 2.2.2 Model Building and Evaluation

The above-described set of 10,653 compounds was randomly divided into a training set (75%) and a validation set (25%), with the ratio of actives/nonactives kept constant (Table S1). The DeepChem module in Maestro (version 2019-2, Schrödinger, NY, USA) served to build a categorical classifier. The maximum training time was limited to 20 min. Five indices were calculated for evaluation. The sensitivity (SE), specificity (SP), enrichment factor (EF), and Matthews’s correlation coefficient (MCC) were defined as follows:
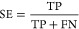

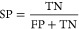

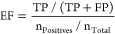

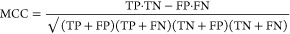


TP (true positive) is the number of active compounds correctly classified, TN (true negative) is the number of inactive compounds correctly classified, FN (false negative) is the number of active compounds that were incorrectly classified, and FP (false positive) is the number of inactive compounds that were incorrectly classified. The fifth index was the area under the receiver operating characteristic curve (AUC).

#### 2.2.3 FooDB Database Virtual Screening

A set of 70,474 compounds was downloaded from the FooDB database (www.foodb.ca, April 2020). The data were curated and prepared for screening using a similar workflow to that described above for the AID884 and AID1851 datasets, with the additional exclusion of inorganic compounds. Compounds from the FooDB were screened to predict their CYP3A4-inhibition potential. Data were further processed post-screening to remove compounds that were outside the applicability domain (APD). Compounds with prediction indices above 0.7 were included in the list of predicted hits. Finally, pesticides and medicinal drugs, traces of which can be sometimes found in food, were manually removed.

#### 2.2.4 Applicability Domain (APD)

The domain of model applicability was calculated to flag compounds in the screening set for which predictions may be unreliable. Fingerprints of compounds in the training and the screening sets were calculated using a chemistry development kit.^17^ The average Tanimoto distance among all pairs of training compounds was calculated. Next, the mean (d) and standard deviation (σ) of a subset of distances that were lower than the average were calculated. The APD was defined as follows:^18^
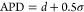


A prediction was considered unreliable when the distance between a screening compound and its nearest neighbor in the training set was greater than the APD.

### 2.3 Coexpression of CYP3A4 and NADPH Cytochrome P450 Reductase in *E. coli*

A bicistronic expression system of CYP3A4 and NADPH cytochrome P450 reductase (POR) was established in *Escherichia coli*. CYP3A4 (UniProt: P08684) and POR (UniProt: P16435) genes were synthesized as *E. coli* codon bias by Genewiz (South Plainfield, NJ, USA). Modification of the CYP3A4 protein sequence included the removal of the initial 10 amino acids at the N terminus, the conversion of the first eight amino acids into MALLLAVF, as described for CYP1A2,^19^ and the addition of a His-tag to the C-terminal region. The pCW-LIC plasmid (#26098) for CYP3A4 cloning was purchased from Addgene (MA, USA). The modified DNA was cloned into the pCW vector via the NdeI and HindIII sites. The pACYC-184 plasmid for POR cloning was generously provided by Gabriel Kaufmann (Biochemistry Department, Tel Aviv University, Israel). The plasmid was restricted at the XbaI and AvaI sites, and the AvaI site was replaced by HindIII. The regulatory elements *tac* promoter, *lac* operator, and *pelB* sequences were synthesized upstream of the POR. The modified DNA was cloned into the pACYP-184 vector via the XbaI and HindIII sites. Plasmids were cotransformed into competent DH5α *E. coli* cells. *E. coli* growth conditions, protein expression, and microsome extraction were as previously described.^20^

### 2.4 CYP3A4 Inhibition

Candidate molecules were dissolved in dimethyl sulfoxide (DMSO) to a stock solution of 10 mM, which was later diluted in 100 mM phosphate buffer (pH 8) to the final concentrations. An equivalent amount of DMSO was used as a control.

CYP3A4 activity was determined using microsomes that were produced from the CYP3A4-recombinant bacteria. Microsomes were preincubated at graduated concentrations (125, 42, 14, 4.6, 1.5, 0.5, and 0.17 μM) of each compound and the fluorogenic Vivid BOMR substrate (Thermo Fisher Scientific, MA, USA), in a black, 96-well, flat-bottom plate at 37 °C for 20 min. An NADPH-generating system (glucose-6-phosphate, glucose-6-phosphate dehydrogenase, and NADP^+^) was then added to initiate the reaction. Fluorescence, indicating the formation of metabolites, was measured at the start of the reaction (*T*_0_) and again 20 min later (*T*_20_) using a Spark microplate reader (Tecan, Männedorf, Switzerland), with a 535 nm wavelength for excitation and 590 nm wavelength for emission. The plate was incubated at 37 °C with shaking during the period in which the reaction occurred. All measurements were performed in triplicate. Inhibition was calculated as the percentages of the corresponding control value in the presence of DMSO alone. IC_50_ values (i.e., the concentration of inhibitor causing a 50% reduction in activity relative to the control) were calculated using nonlinear regression analysis with Prism version 8.3.0 (GraphPad Software, CA, USA).

## 3 Results

### 3.1 DeepChem Activity Model

We used published data on the potency of CYP3A4 inhibition by numerous compounds to build a prediction model. This model was used to screen natural compounds present in food. The AUC of the receiver operating characteristic curve was 0.97 (the full curve is shown in Figure S1). A threshold index for the model is required to define compounds as active or inactive. Here, we selected a threshold of 0.7 to balance the trade-off between high specificity and a high number of hit molecules. The SP of the model was found to be 0.997, its SE was 0.551, the MCC was 0.7, and the EF was 5.871 using the threshold and validating with an external validation set (Table S2).

### 3.2 Virtual Screening of the FooDB Database

Following the curation process, the FooDB database contained 68,900 unique compounds. The curated database was screened using the new model, resulting in the identification of 136 compounds as active (index ≥ 0.7). Postscreening curation (see Section 2) yielded 115 hits (Table 1); the abilities of 84 of these compounds to inhibit CYP3A4 have not yet been characterized. Twenty-three compounds were previously recognized as CYP3A4 inhibitors (IC_50_ ≤ 20 μM), seven of which were shown to be highly potent with IC_50_ ≤ 1 μM. Another eight compounds have been shown to be weak inhibitors or inactive (IC_50_ > 20). This small data sample has a TP/TF ratio of ∼3.

**Table 1:** Compounds Predicted by the Classifier to Be CYP3A4 Inhibitors

FooDB ID	CAS no.	name	MW	AlogP	HBA	HBD	pred. index	lit. IC_50_ (μM)	reference
FDB022708	52-53-9	verapamil	455.6	4.0	5	0	0.93	2	(21)
FDB016335	961-29-5	isoliquiritigenin	256.3	2.8	4	3	0.92	14.51	(22)
FDB000449	94-62-2	piperine	285.3	2.8	3	0	0.91	17	(23)
FDB000444	25924-78-1	piperyline	271.3	2.3	3	0	0.89	3.6	(23)
FDB011523	485-61-0	graveoline	279.3	3.8	3	0	0.89		new
FDB015236	342371-98-6	pipercyclobutanamide B	596.7	5.2	6	0	0.89		new
FDB015235	479068-69-4	pipercyclobutanamide A	570.7	4.7	6	0	0.88		new
FDB020288	500757-86-8	dipiperamide E	570.7	4.7	6	0	0.88	0.63	(23)
FDB000678	480-41-1	naringenin	272.3	2.3	5	3	0.87	8.8	(24)
FDB011479	6035-40-1	gnoscopine	413.4	3.0	7	0	0.87		new
FDB016658	1668-33-3	demethoxykanugin	326.3	3.6	6	0	0.87		new
FDB018430	112448-63-2	5-methoxyhinokinin	384.4	3.6	7	0	0.86		new
FDB020287	500757-85-7	dipiperamide D	596.7	5.2	6	0	0.86	0.79	(23)
FDB018555	69239-53-8	5-geranyloxy-8-methoxy-psoralen	368.4	5.3	5	0	0.86		new
FDB012066	23512-46-1	piperanine	287.4	3.2	3	0	0.86		new
FDB011280	101751-72-8	isoyatein	400.4	3.8	7	0	0.85		new
FDB014637	751-03-1	obacunone	454.5	2.1	7	0	0.85	20.9	(25)
FDB011672	94-59-7	safrole	162.2	2.6	2	0	0.84	>100	(24)
FDB019057	432041-19-5	dipiperamide A	570.7	4.8	6	0	0.84	0.18	(23)
FDB003977	2543-94-4	phellopterin	300.3	3.5	5	0	0.83	1	(26)
FDB018269	117137-65-2	4,5-dihydropiperyline	273.3	2.7	3	0	0.83		new
FDB011395	15358-38-0	oxonantenine	335.3	2.8	6	0	0.83		new
FDB020111	155416-22-1	simulanoquinoline	618.7	6.4	7	0	0.83		new
FDB001651	3187-53-9	anhydropisatin	296.3	3.3	5	0	0.83		new
FDB005955	477-47-4	picropodophyllin	414.4	2.1	8	1	0.83		new
FDB011580	120834-89-1	lambertine	337.4	3.2	4	0	0.82		new
FDB019058	432041-21-9	dipiperamide C	556.6	4.2	6	0	0.82	0.48	(23)
FDB012402	1242-81-5	dehydroneotenone	336.3	3.3	6	0	0.82		new
FDB001446	133067-72-8	(3*R*)-sophorol	300.3	2.2	6	2	0.81		new
FDB012745	583-34-6	piperettine	311.4	3.2	3	0	0.81		new
FDB017387	93767-25-0	jangomolide	468.5	1.4	8	0	0.81		new
FDB018040	117137-67-4	brachyamide B	327.4	4.1	3	0	0.81		new
FDB012281	1006528-8	dolineone	336.3	2.7	6	0	0.81		new
FDB013402	205,115-74-8	lansiumarin B	370.4	4.1	6	1	0.81		new
FDB005049	1180-71-8	limonin	470.5	0.9	8	0	0.81	19.1	(27)
FDB002789	521-32-4	bilobetin	552.5	6.1	10	5	0.81		new
FDB001536	2196-14-7	7,4′-dihydroxyflavone	254.2	3.3	4	2	0.81		new
FDB015348	107534-93-0	macelignan	328.4	5.2	4	1	0.81	>100	(28)
FDB020938	154490-59-2	1,2-dimethoxy-13-methyl-[1,3]benzodioxolo[5,6-*c*]phenanthridine	347.4	3.9	5	0	0.80		new
FDB011383	36285-03-7	dehydroaporheine	277.3	3.7	2	0	0.80		new
FDB014514	1063-77-0	nomilin	514.6	1.6	9	0	0.80	10.4	(25)
FDB018057	12751-00-7	cicerin	330.3	2.2	7	2	0.80		new
FDB014460	101560-02-5	dukunolide D	468.5	1.4	8	2	0.80		new
FDB011902	481-46-9	ginkgetin	566.5	6.3	10	4	0.80		new
FDB021029	159465-79-9	8-methyldihydrochelerythrine	363.4	4.3	4	0	0.80		new
FDB012143	548-19-6	isoginkgetin	566.5	6.3	10	4	0.80		new
FDB014461	101559-97-1	dukunolide E	484.5	0.2	9	2	0.79		new
FDB002651	60132-69-6	betagarin	328.3	2.8	6	0	0.79		new
FDB018039	117137-68-5	(2E,4E,8E)-piperamide-C9:3	325.4	3.7	3	0	0.79	4.2	(23)
FDB002766	480-44-4	acacetin	284.3	3.3	5	2	0.79	1.2	(24)
FDB006932	437-64-9	genkwanin	284.3	3.3	5	2	0.79		new
FDB002709	28768-44-7	(+)-12α-hydroxypachyrrhizone	382.3	2.0	8	1	0.79		new
FDB020627	107584-38-3	dehydropipernonaline	339.4	4.1	3	0	0.79		new
FDB017322	77053-35-1	5′-methoxybilobetin	582.5	6.1	11	5	0.78		new
FDB013739	20086-05-9	diosbulbin A	376.4	0.5	7	1	0.78		new
FDB018042	112448-68-7	(2E,6E)-piperamide-C7:2	299.4	3.2	3	0	0.78		new
FDB014182	223558-40-5	vitisifuran B	904.9	11.0	12	9	0.78		new
FDB018566	105866-30-6	epoxybergamottin	354.4	4.1	5	0	0.77	1.5	(29)
FDB000586	3736-83-2	erosnin	320.3	3.3	6	0	0.77		new
FDB013340	223591-28-4	vitisifuran A	904.9	10.9	12	10	0.77		new
FDB003953	482-45-1	isoimperatorin	270.3	3.5	4	0	0.77	2.7	(26)
FDB015532	90–29-9	pseudobaptigenin	282.2	2.9	5	1	0.77		new
FDB015547	14348-21-1	cnidicin	354.4	5.0	5	0	0.77		new
FDB014654	607-91-0	myristicin	192.2	2.6	3	0	0.76	43.2	(24)
FDB001853	1009344-00-6	kuguacin B	418.5	3.8	4	1	0.76		new
FDB006431	482-48-4	isobergapten	216.2	2.1	4	0	0.76	>100	(26)
FDB001777	23740-25-2	oxoxylopine	305.3	2.9	5	0	0.76		new
FDB019861	267428-36-4	paradisin C	726.8	8.0	11	2	0.76	1	(30)
FDB011388	70560-83-8	isodomesticine	325.4	3.1	4	1	0.76		new
FDB014401	3264-90-2	deacetylnomilin	472.5	1.3	8	1	0.75	63.2	(25)
FDB019053	1240562-92-8	argenteane	654.8	10.0	8	2	0.75		new
FDB011478	521-40-4	narcotoline	399.4	2.8	7	1	0.75		new
FDB011746	223591-26-2	viniferifuran	452.5	6.0	6	5	0.75		new
FDB000714	131-12-4	pimpinellidine	246.2	2.1	5	0	0.75		new
FDB019556	37687-34-6	xi-8-acetonyldihydrosanguinarine	389.4	3.4	5	0	0.75		new
FDB018295	101140-06-1	3,8″-biapigenin	538.5	5.8	10	6	0.75	0.082	(31)
FDB097289	84,870-54-2	gnetin C	454.5	5.5	6	5	0.75		new
FDB023165	2086-83-1	berberine	336.4	3.7	4	0	0.75	48.9	(32)
FDB001445	21495-84-1	2-hydroxypseudobaptigenin	298.2	2.7	6	2	0.74		new
FDB097354	153-18-4	rutin	678.7	8.6	8	7	0.74	45	(33)
FDB012178	259244-41-2	isopiperolein B	343.5	4.7	3	0	0.74		new
FDB016343	60857-34-3	2,3-dihydro-5,5′,7,7′-tetrahydroxy-2-(4-hydroxyphenyl)[3,8′-bi-4*H*-1-benzopyran]-4,4′-dione	448.4	3.3	9	5	0.74		new
FDB002183	30505-89-6	piperolein B	343.5	4.7	3	0	0.74	1.4	(23)
FDB002417	520-30-9	norartocarpetin	286.2	2.8	6	4	0.73		new
FDB013637	989-23-1	desoxylimonin	454.5	1.7	7	0	0.73		new
FDB016542	107585-75-1	dihydroeucomin	316.3	2.6	6	3	0.73		new
FDB021208	165883-77-2	r-viniferin	906.9	10.5	12	9	0.73		new
FDB016339	51828-10-5	2′-methylisoliquiritigenin	270.3	3.0	4	2	0.73		new
FDB018041	62510-52-5	tricholein	329.4	4.3	3	0	0.73	2.8	(23)
FDB002688	28617-71-2	13α-hydroxydolineone	352.3	2.1	7	1	0.73		new
FDB000319	62218-13-7	δ-viniferin	454.5	5.5	6	5	0.73		new
FDB019636	462636-73-3	gnemonol A	696.7	7.6	10	8	0.73		new
FDB015723	67567-13-9	diosbulbin H	418.5	1.7	7	1	0.73		new
FDB010679	75022-26-3	dihydroretrofractamide B	357.5	5.6	3	1	0.72		new
FDB022660	3735-01-1	aminoparathion	261.3	2.6	3	1	0.72		new
FDB013871	111004-32-1	isocyclocalamin	502.6	0.9	9	1	0.72		new
FDB093560	16851-21-1	morelloflavone	556.5	4.8	11	7	0.72		new
FDB015548	2035-15-6	maackiain	284.3	2.4	5	1	0.72	52.91	(34)
FDB013403	205115-73-7	lansiumarin A	352.4	4.3	5	0	0.72		new
FDB011386	2466-42-4	neolitsine	323.3	3.1	4	0	0.72		new
FDB002141	517-66-8	dicentrine	339.4	3.3	4	0	0.72		new
FDB016352	16266-97-0	3,5,6-trimethoxyflavone	312.3	3.8	5	0	0.72		new
FDB000610	480-43-3	isosakuranetin	286.3	2.5	5	2	0.71	4.3	(35)
FDB011387	41787-55-7	cryptodorine	309.3	2.6	4	0	0.71		new
FDB000082	487-52-5	butein	272.3	2.5	5	4	0.71		new
FDB014643	74751-39-6	cyclocalamin	502.6	0.9	9	1	0.71		new
FDB015719	20086-06-0	diosbulbin B	344.4	0.9	6	0	0.71		new
FDB012616	530-22-3	egonol	326.3	3.8	5	1	0.71		new
FDB011341	168037-22-7	miyabenol C	680.7	8.0	9	7	0.71		new
FDB001472	2957-21-3	sakuranetin	286.3	2.5	5	2	0.71	<10	(36)
FDB011998	1983-72-8	medicagol	296.2	2.9	6	1	0.71		new
FDB019458	485794-76-1	pipertipine	329.4	4.3	3	0	0.71		new
FDB014215	313485-83-5	ginsenoyne N	462.7	8.6	2	0	0.71		new
FDB001753	20979-50-4	7,4’-dimethoxyflavone	282.3	3.8	4	0	0.71		new
FDB011382	5890-28-8	cassythicine	325.4	3.1	4	1	0.70		new

### 3.3 Criteria for Candidate Intestinal Inhibitors

To highlight compounds that are especially relevant as intestinal CYP3A4 inhibitors, we applied additional filtration criteria based on Lipinski’s Rule of five (LRO5):^37^ molecular weight (MW) greater than 500, lipophilicity [expressed as the calculated ratio of octanol solubility to aqueous solubility (ClogP)] greater than five, more than five H-bond donors, and more than 10 H-bond acceptors. These rules help us to focus on compounds with a low probability of being absorbed through the intestinal epithelium and reaching the blood. Such compounds will be effective in modifying intestinal CYP3A4 activity. Applying the nonabsorbance criteria to the 115 hits from the model narrowed the list to 17 candidate compounds violating two or more of the parts of the LRO5 (Table 2). Three compounds, paradisin C, dipiperamide D, and 3,8″-biapigenin have indeed been reported to be highly potent CYP3A4 inhibitors (with IC_50_ values of 1, 0.79, and 0.08 μM, respectively).^23,30,31^ Rutin has been reported to be a weak CYP3A4 inhibitor (IC_50_ = 45 μM).^33^ To the best of our knowledge, the CYP3A4-inhibition capacities of the remaining 13 compounds have not been reported in the scientific literature.

**Table 2:**
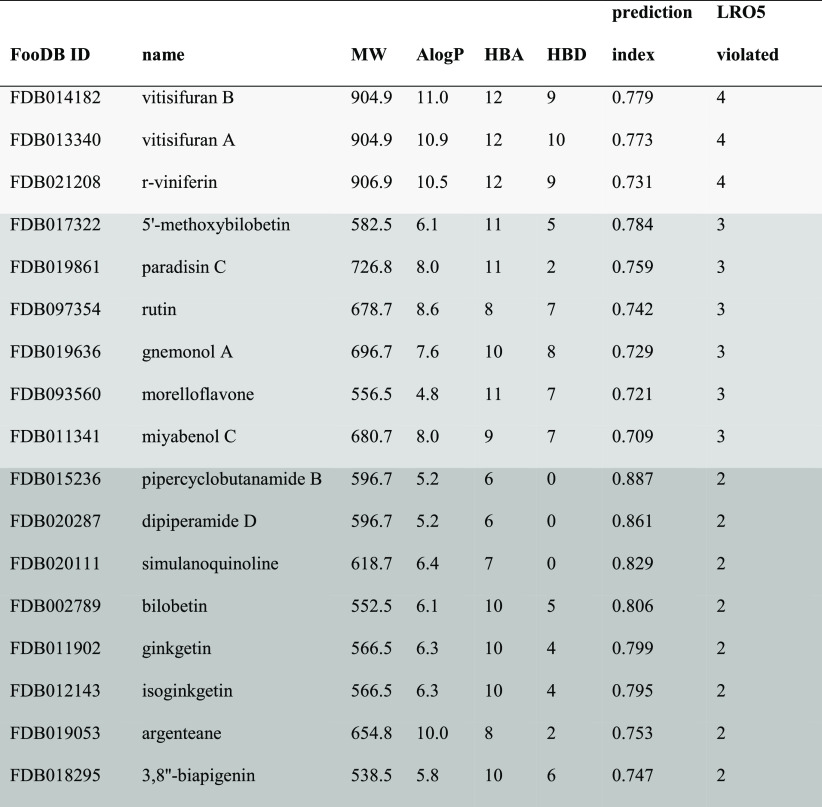
Hits that Met the Intestinal Nonpermeability Criteria^a^

a MW—molecular weight, AlogP—calculated octanol–water partition coefficient, HBA—hydrogen bond acceptors, HBD—hydrogen bond donors, LRO5—Lipinski’s rules of five for intestinal absorption.

### 3.4 Analysis of Chemical Families

**Figure 2:**
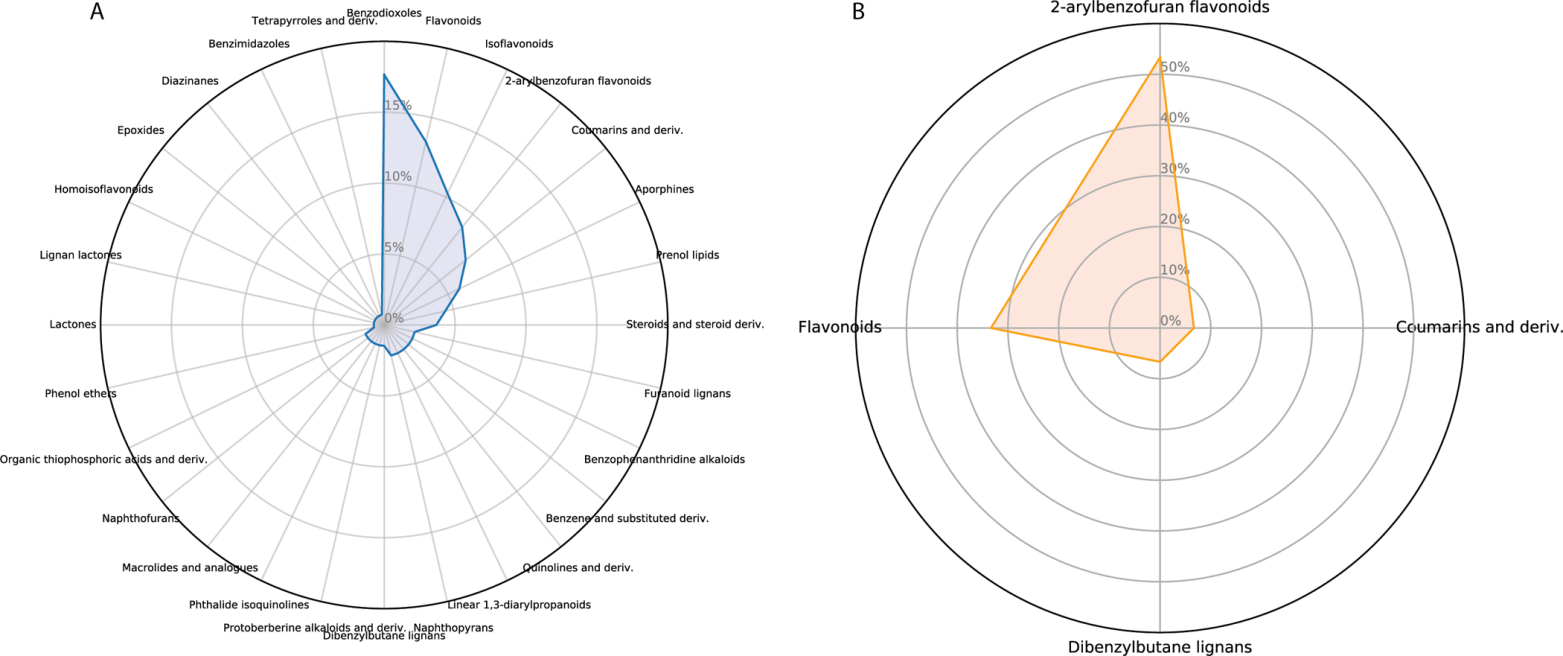
Radar presentation of the chemical families in the FooDB database. (A) Compounds predicted by the model as inhibitors. (B) Compounds violating two or more parts of Lipinski’s Rule of five.

CYP3A4’s large and promiscuous active site allows both structurally and chemically diverse compounds to bind as ligands and as modifiers. Here, we analyzed which chemical families are rich in CYP3A4 inhibitors. The most abundant chemical families among FooDB-derived predicted inhibitors are presented in Figure 2; that presentation suggests a split between numerous diverse chemical families (Figure 2A). The set of potential inhibitors includes flavonoids, coumarins, aporphines, and steroids, and less than 20% of the predicted inhibitors belong to the main group of benzodioxoles. The nonpermeable compounds in this database are mostly flavonoids (Figure 2B).

### 3.5 Analysis of Foods Containing CYP3A4 Inhibitors

**Figure 3:**
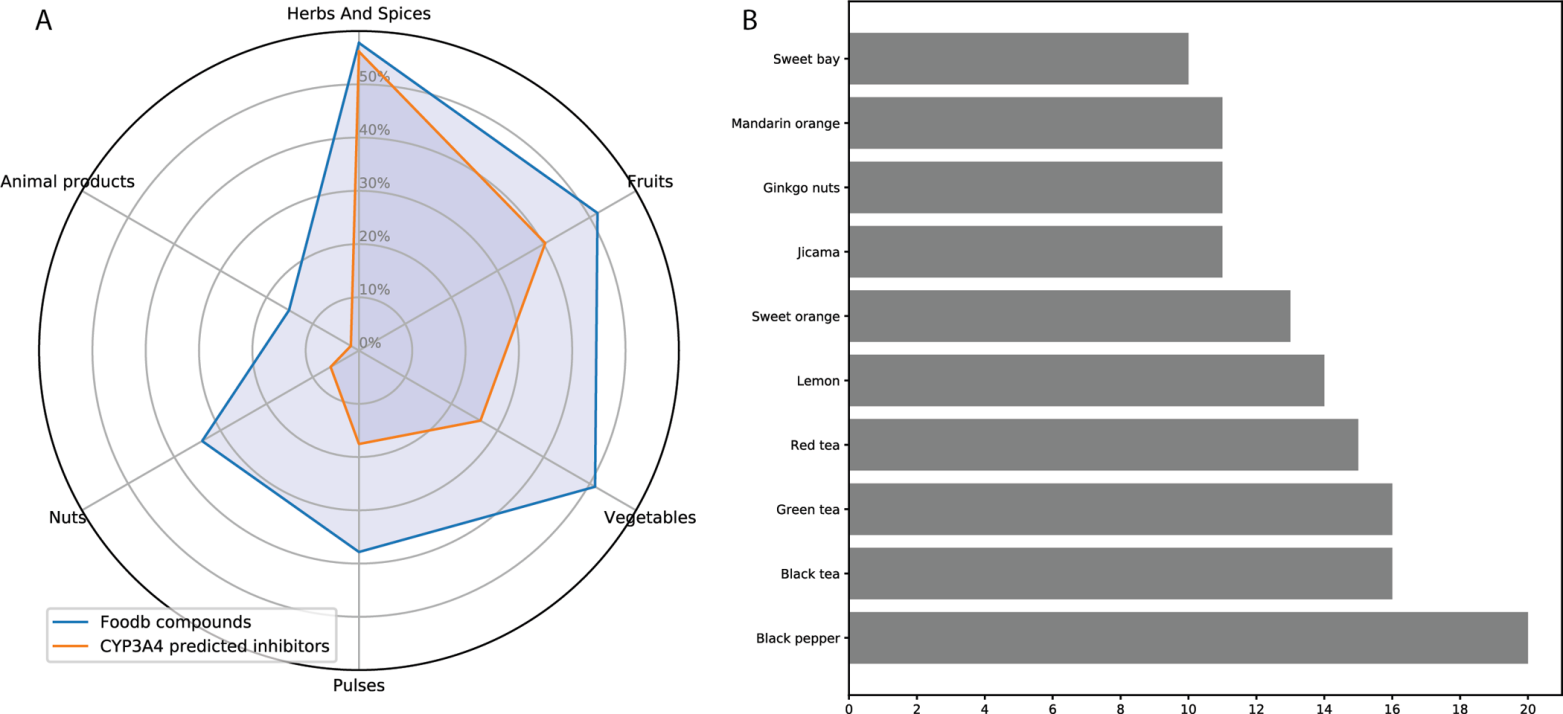
Foods containing compounds predicted by FooDB to inhibit CYP3A4. (A) Analysis by food groups. The proportion of compounds related to the group in the total number of compounds is shown. Some compounds were assigned to more than one group. (B) Top 10 foods containing CYP3A4 inhibitors.

Numerous secondary metabolites are produced by edible plants, as has been well documented for plant-based foods. Indeed, more than 50% of all compounds in FooDB are classified in the Herbs and Spices group (Figure 3A), with the Fruits group containing slightly more compounds than the Vegetables group. Only 15% of all FooDB compounds are animal-derived food products. The Herbs and Spices group also includes the largest number of predicted inhibitors, again followed by the Fruits and Vegetables groups. Among the top 10 foods that contain a large variety of inhibitors, black pepper (*Piper nigrum*) leads with 20 different candidate compounds, of which only five were previously acknowledged as inhibitors. Other food families are herbal teas, citrus fruit, the unique *Ginkgo biloba*, jicama (*Pachyrhizus erosus*), and sweet bay (*Laurus nobilis*), each with more than 10 different compounds predicted to be CYP3A4 inhibitors (Figure 3B). However, this list should be considered with caution, as while FooDB is the most comprehensive resource on food constituents, quantitative data regarding the content of compounds in the foods are available for only a small number of compounds. Exploring these data (Table S3), it is worth paying attention to high-concentration compounds in some foods: Mexican oregano (*Lippia graveolens*) is rich in sakuranetin (prediction index = 0.71, experimental IC_50_ < 10,^36^ and content = 93 mg/100 g), and black pepper contains high levels of both piperine (prediction index = 0.91, experimental IC_50_ = 17.2,^23^ and content = 5350 mg/100 g) and piperettine (prediction index = 0.81; content = 525 mg/100 g). Myristicin (prediction index = 0.76; experimental IC_50_ < 43^24^) appears in sufficient concentrations in parsnip (*Pastinaca sativa*), parsley (*Petroselinum crispum*), mace (*Myristica fragrans*), caraway (*Carum carvi*), and carrot (*Daucus carota*) (content levels: 42 g/100 g, 1.7 g/100 g, 0.68 g/100 g, 24 mg/100 g, and 1.7 mg/100 g, respectively). A serving of red wine (150 mL) contains 1 mg of viniferin (prediction index = 0.73).

### 3.6 In Vitro Inhibition Capacity

**Figure 4:**
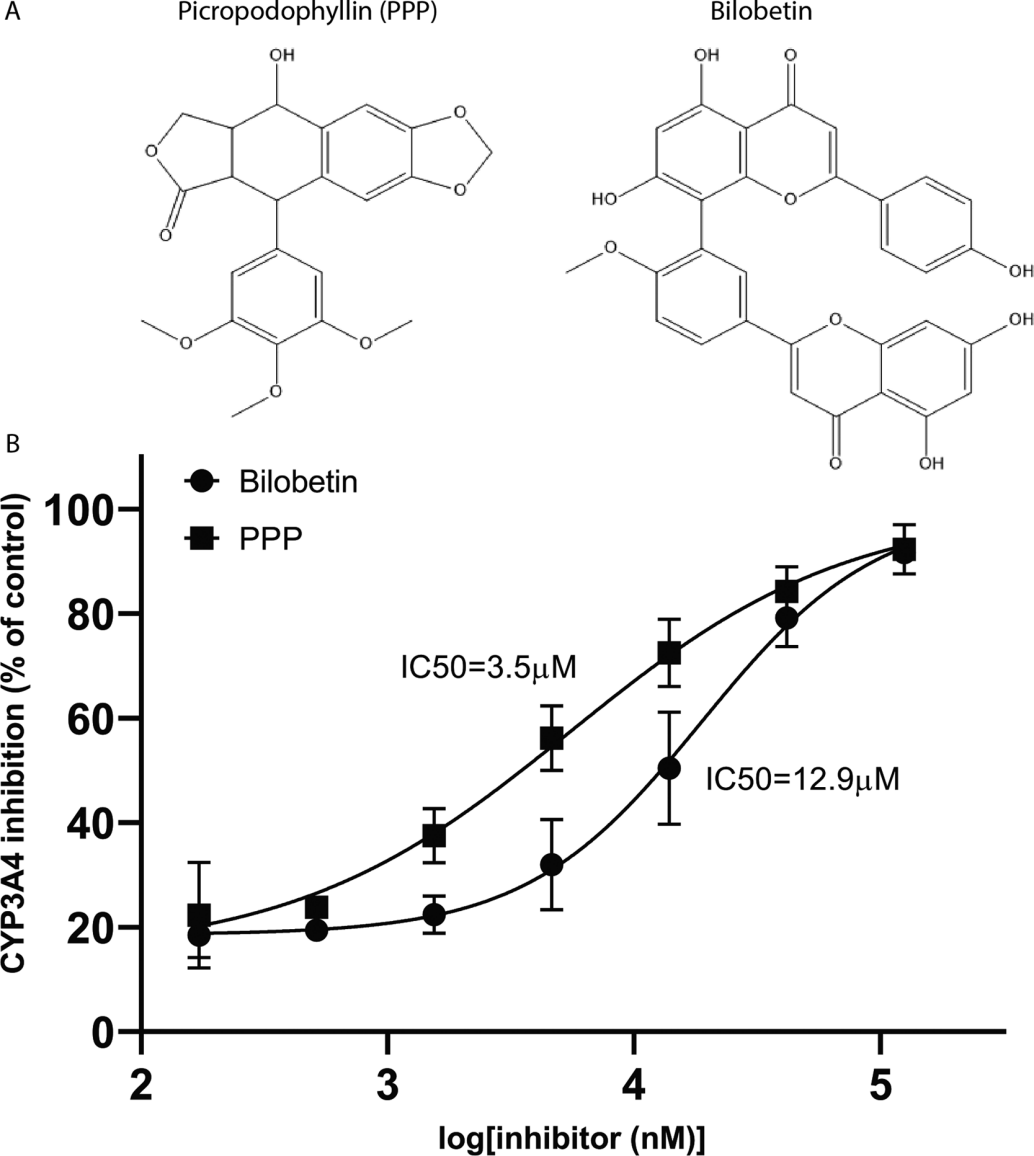
CYP3A4 inhibition by representative compounds. (A) Chemical structures. (B) Results are expressed as the mean ± SE of the values from three independent experiments. IC_50_ values were calculated using nonlinear regression (*R*^2^ = 0.913 for bilobetin and *R*^2^ = 0.903 for PPP).

Bilobetin and picropodophyllin (PPP) were predicted by the model presented here to be potent inhibitors of CYP3A4, with prediction indices of 0.81 and 0.83, respectively. To further confirm the prediction and the validity of the model, we tested the inhibitory potency of these compounds using recombinant CYP3A4 expressed in *E. coli* microsomes and a fluorogenic CYP3A4-specific substrate. Both compounds were indeed shown to be potent inhibitors of CYP3A4, with an IC_50_ of 3.5 μM for PPP and an IC_50_ of 12.9 μM for bilobetin (Figure 4).

## 4 Discussion

The model developed and presented here was designed to accurately and rapidly screen and classify compounds in food as either potent inhibitors of CYP3A4 activity or as inactive compounds (i.e., noninhibiting). Such models capable of screening numerous compounds while taking into account intestinal activity are required for dealing with the overwhelming variety of dietary compounds. The model achieved an MCC score of 0.7. The MCC score (a value between −1 and + 1) measures the correlation coefficient between the observed and predicted binary classifications. MCC is considered to be the best tool for evaluating classifiers with classes of very different sizes.^38^ This value indicates the successful classification of compounds as either active or inactive.

Natural products stand out for their enormous structural and physicochemical diversity.^39^ This great diversity is expressed as a large chemical space (Figure S3A). The APD specifies the scope of a prediction model, defining the model’s limitations with respect to its physicochemical domain. If an external compound is beyond the defined scope of a given model, it is considered to be outside that model’s applicability domain and cannot be associated with a reliable prediction. The model was trained with a chemically diverse array of compounds, which resulted in a wide APD and made it applicable to the heterogeneous group of phytochemicals. A principal component analysis verified that indeed the predicted active compounds in FooDB share the chemical space of the truly active compounds from the PubChem dataset (Figure S3B).

Using a threshold of 0.7 for the prediction index, we observed higher specificity values than sensitivity values, indicating that the proposed model yields fewer false positives than false negatives. This is in line with our goal to accurately highlight candidate CYP3A4 inhibitors. The specificity, MCC, and AUC of our new model are close to and somewhat higher than those of other previously published quantitative structure–activity relationship and machine-learning models.^12,34^

To the best of our knowledge, this is the first time that LRO5 has been used to mark candidate compounds for their local inhibition activity in the intestine. Here, with a major focus on compounds from food, we directed our attention to nonabsorbed compounds that are usually screened out by LRO5. The importance of the inhibition of intestinal CYP3A4 activity has been recognized by many^6,7,40−43^ and highlights interest in potential inhibitors of the intestinal CYPs.

The TP/FP ratio of our model was 32.23, suggesting that out of 100 top-ranked molecules, only ∼3 would be falsely classified as active (TP/FP = 97/3). One hundred and fifteen compounds were classified as inhibitors, of which we were able to find in the literature experimental data for only 31. An additional two compounds were tested in vitro in this work. Of these 33 compounds, 25 were found to be potent inhibitors, indicating a TP/FP ratio of 3.1. The calculated TP/FP value, which is lower than that predicted by the model, is still higher than the initial active/nonactive ratio of the curated AID data (nonactives here include both the 8893 true inactives and 7149 inconclusive compounds of the curated dataset), which is 0.11 (actives/nonactives = 1760/16042). In accord with this value, we would expect to find only 10 inhibitors in a random subset of 100 molecules. Applying our model to the FooDB dataset, we have presumably found 75 inhibitors among the top 100 compounds, indicating a 7.5 times enrichment and a successful classifier model.

Computational models are as good as the quality of the data on which they are based. To build and externally test our model, we used high-throughput in vitro data based on a uniform single assay. The data regarding the inhibition of CYP3A4 by natural products are a collection of previously published results, achieved by various, separately executed assays. This may explain the difference between the calculated TP/FP ratios of the model based on the test set and the TP/FP ratio from the FooDB collection of experimental data.

Applying the model yielded 84 newly classified compounds as inhibitors. In vitro testing of two compounds demonstrated the validity of the model. Still, using a sensitivity of 0.55 implies that some truly active compounds were not classified as such (Figure S2) and that there might be more dietary inhibitors yet to be discovered.

A substantial hurdle in analyzing the in vitro and in vivo inhibition of CYP3A4 by natural products is the limited to nonexistent commercial availability of those natural products. The compounds assayed here, such as bilobetin, a biflavonoid from *Gingko biloba*, and PPP, a cyclolignan alkaloid mainly found in the mayapple plant (*Podophyllum peltatum*), were selected not only due to their predicted inhibition capacity and their varied structures but also due to their commercial availability. This is the first report of the effects of these compounds on CYP3A4 activity. The IC_50_ values of PPP and bilobetin are 3.5 and 12.9 μM, respectively. The best-known FDI, also known as “the grapefruit effect,” is caused by a group of compounds with IC_50_ values that are within that range.^29,44^ The “grapefruit effect” was shown to be a critical contributor to failure in medical treatments. This dictates strict medical guidelines, demonstrating the biological relevance of these IC_50_ values.^45,46^ Bilobetin is a flavonoid thus representing a major group of natural products heavily consumed in most diets and particularly as traditional medicine remedies coadministrated with conventional drugs. Mean flavonoid intake is 330 mg/day in the United States and 430 mg/day in Europe.^47^ This amount, which is generally considered safe, should be reassessed in light of the new data regarding CYP3A4-inhibition and potential impaired phase I metabolism and even food–drug interactions that may be caused during drug administration. Bilobetin exhibits various pharmacological properties, such as antioxidant, anticancer, antibacterial, antifungal, anti-inflammatory, and antiviral properties; it also promotes osteoblast differentiation.^48^ PPP has recently received attention due to its anticancer activity as an inhibitor of the insulin-like growth factor receptor (IGF-IR).^49^ These properties make bilobetin and PPP good candidates for drug development or as traditional supplements to conventional medicine. Considering this, experimental evidence for their ability to inhibit intestinal CYP3A4 is especially relevant.

Seventeen molecules in the list of predicted inhibitors violate two or more of the LRO5. These molecules are of special interest due to the reduced absorption expected into the bloodstream and their projected inhibition of intestinal CYP3A4, which might go unnoticed in experiments conducted on rodents.^1,50^ In humans, intestinal CYP3A4 accounts for 80% of the total P450 intestinal content, but CYP3A4 is not present in rodents’ intestines. The passage of digested food through the large intestine in humans can take up to 24 h or more,^51^ a sufficient amount of time for substantial amounts of natural compounds to induce functional alteration of intestinal CYP3A4.

Herbal supplements and medicinal products have been used for centuries to support and maintain physiological functions and to combat ailment. The consumption of these products has increased tremendously over the past three decades with at least 80% of the world’s population relying on them for at least partial primary healthcare.^52^ For instance, *Gingko biloba* and green tea, found here to contain over 10 different predicted inhibitors of CYP3A4, are among the most popular herbal supplements used to strengthen wellness.^53^ Piperine, yet another newly predicted inhibitor with a prediction index of 0.9, is often added to curcumin and to coenzyme Q10, to increase their bioavailability. Our findings also support the well-known inhibitory effect of citrus, especially grapefruit.^54^

Along with their consumption, the awareness of the potential hazards of botanical supplements has increased in recent years.^4^ However, the limited data about herb–drug interactions have led to limited conclusions. A comprehensive review published a few years ago in the *Annual Review of Pharmacology and Toxicology*(4) mentioned five botanical supplements that interact with medical treatment via CYP3A4. A review published recently^54^ addresses many more fruits and vegetables that might impair toxin and xenobiotics metabolism in the intestine and possibly cause deleterious FDI. Data for specific compounds that might be responsible for such interactions are limited. Using virtual screening provided a significant addition to the data and interpretations that are available to the scientific community and to nutrition and clinical professionals.

## Supporting Information Available

(Figure S1) Receiver-operating-characteristic (ROC) curve of the validation set; (Figure S2) distribution of truly active and inactive compounds by prediction indices; (Figure S3) 2D chemical space presented by the second principal component (PC) plotted against the first PC based on physicochemical descriptors; (Table S1) composition of training and validation sets; and (Table S2) model performance with cut-offs at different indices (PDF)
(Table S3) Full data table of predicted CYP3A4 inhibitors and their content in foods (XLSX)
