# Supplementary material for: Dietary Inhibitors of CYP3A4 Are Revealed Using Virtual Screening by Using a New Deep-Learning Classifier
Source: J Agric Food Chem. 2022 Feb 1;70(8):2752–61. doi: 10.1021/acs.jafc.2c00237 (PMC8895463; doi:10.1021/acs.jafc.2c00237)
Supplement: Supplementary file 1 — jf2c00237_si_001.pdf [file jf2c00237_si_001.pdf]

Supporting Information

Dietary inhibitors of CYP3A4 are revealed using virtual screening by using a new deep learning classifier

Yelena Guttman, Zohar Kerem*

Institute of Biochemistry, Food Science and Nutrition, The Robert H. Smith Faculty of Agriculture, Food and Environment, The Hebrew University of Jerusalem.

POB 12 Rehovot 76100, Israel

*Correspondence to:

E-mail: zohar.kerem@mail.huji.ac.il

Tel: 972-8-9489278;

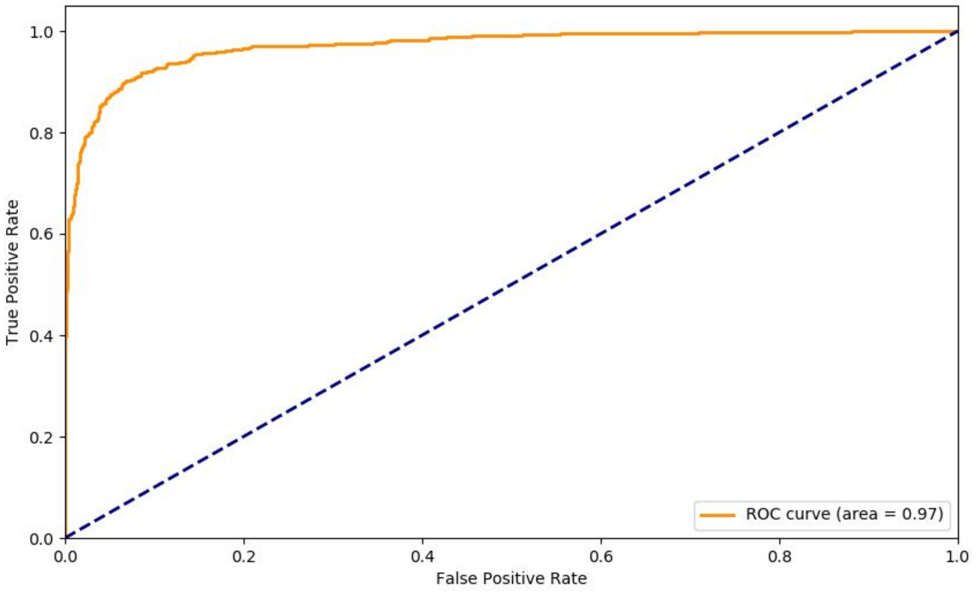

Figure S1: Receiver-operating-characteristic (ROC) curve of the validation set. The continuous and the dashed lines represent our classifier and a random classifier, respectively.

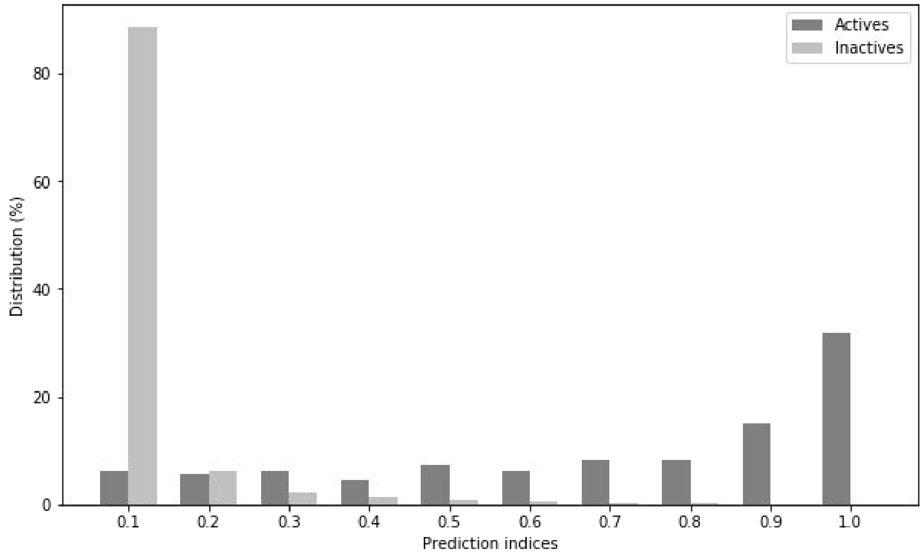

Figure S2: Distribution of truly active and inactive compounds by prediction indices. Compounds in the validation set were scored by the classifier. The scores (indices), in a range of 0-1, indicate the probability of a compound to be CYP3A4 inhibitors.

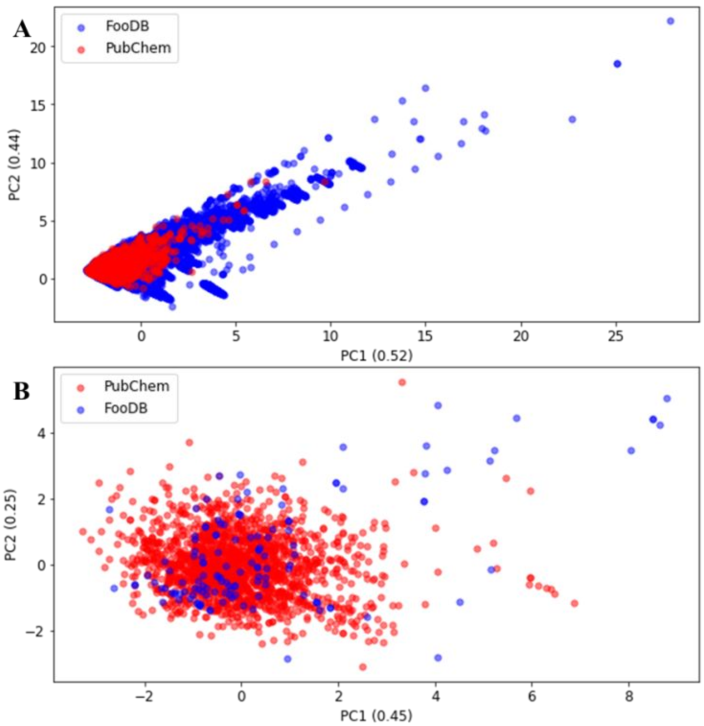

Figure S3: 2D chemical space presented by the second principal component (PC) plotted against the first PC based on physicochemical descriptors. (A) All compounds used for model building (PubChem) and screening (FooDB). (B) Active compounds from PubChem and FooDB databases. The four descriptors of LRO5: molecular weight, lipophilicity (AlogP), number of H-bond donors, and number of H-bond acceptors were calculated using Canvas (Schrödinger, NY, USA). Principal components were calculated using the Scikit-learn package in Python 3.7.4. One outlier compound (PubChem SID 26748902) was omitted.

Table S1: Composition of training and validation sets

	Training set	Validation set	Total
**Actives**	1350	410	1760
**Inactives**	6639	2254	8893
**Total**	7989	2664	10653

Table S2: Model performance with cut-offs at different indices.

Index	TP	FP	TN	FN	MCC	SE	SP	EF	TP/FP
**0**	**410**	**2254**	**0**	**0**	**0**	**1**	**0**	**0.932**	**0.18**
**0.1**	384	260	1994	26	0.6921	0.9366	0.8847	3.609	1.48
**0.2**	361	121	2133	49	0.7750	0.8805	0.9463	4.533	2.98
**0.3**	335	72	2182	75	0.7875	0.8171	0.9680	4.982	4.65
**0.4**	316	44	2210	94	0.7930	0.7707	0.9805	5.313	7.18
**0.5**	286	29	2225	124	0.7652	0.6976	0.9871	5.500	9.86
**0.6**	260	15	2239	150	0.7442	0.6341	0.9933	5.723	17.33
**0.7**	226	7	2247	184	0.7001	0.5512	0.9969	5.871	32.29
**0.8**	192	2	2252	218	0.6491	0.4683	0.9991	5.990	96
**0.9**	130	1	2253	280	0.5284	0.3171	0.9996	6.007	130
**1**	0	0	2254	410	-	0	1	-	-
